# Supplementary material for: MetaRibo-Seq measures translation in microbiomes
Source: Nat Commun. 2020 Jun 29;11:3268. doi: 10.1038/s41467-020-17081-z (PMC7324362; doi:10.1038/s41467-020-17081-z)
Supplement: Supplementary file 10 — Supplementary Data 7 [file 41467_2020_17081_MOESM10_ESM.zip › File2/Confidence_High_Taxonomy/180855_out.krona.html]

Javascript must be enabled to view this page.

members
magnitude
magnitudeUnassigned
count
unassigned
taxon
rank

180855\_out

9

superkingdom
9
2

phylum
1239
9

class
9
186801

9
186802
order

family
9
186803

841
8
genus


SRS014613\_contig\_number\_21456SRS015190\_contig\_number\_15873SRS015960\_contig\_number\_16496SRS024388\_contig\_number\_13257SRS055017\_contig\_number\_37273SRS142503\_contig\_number\_contig-100\_1250.299448SRS143070\_contig\_number\_contig-100\_187.104800SRS149181\_contig\_number\_contig-100\_344.185623
species
8
166486

1
572511
genus


SRS050422\_contig\_number\_27015
species
1
1262758
